# Supplementary material for: Complex Autism Spectrum Disorder with Epilepsy, Strabismus and Self-Injurious Behaviors in a Patient with a De Novo Heterozygous POLR2A Variant
Source: Genes (Basel). 2022 Mar 7;13(3):470. doi: 10.3390/genes13030470 (PMC8955435; doi:10.3390/genes13030470)
Supplement: Supplementary file 1 [file genes-13-00470-s001.zip › genes-1594767-supplementary.pdf]

Supplementary Materials:

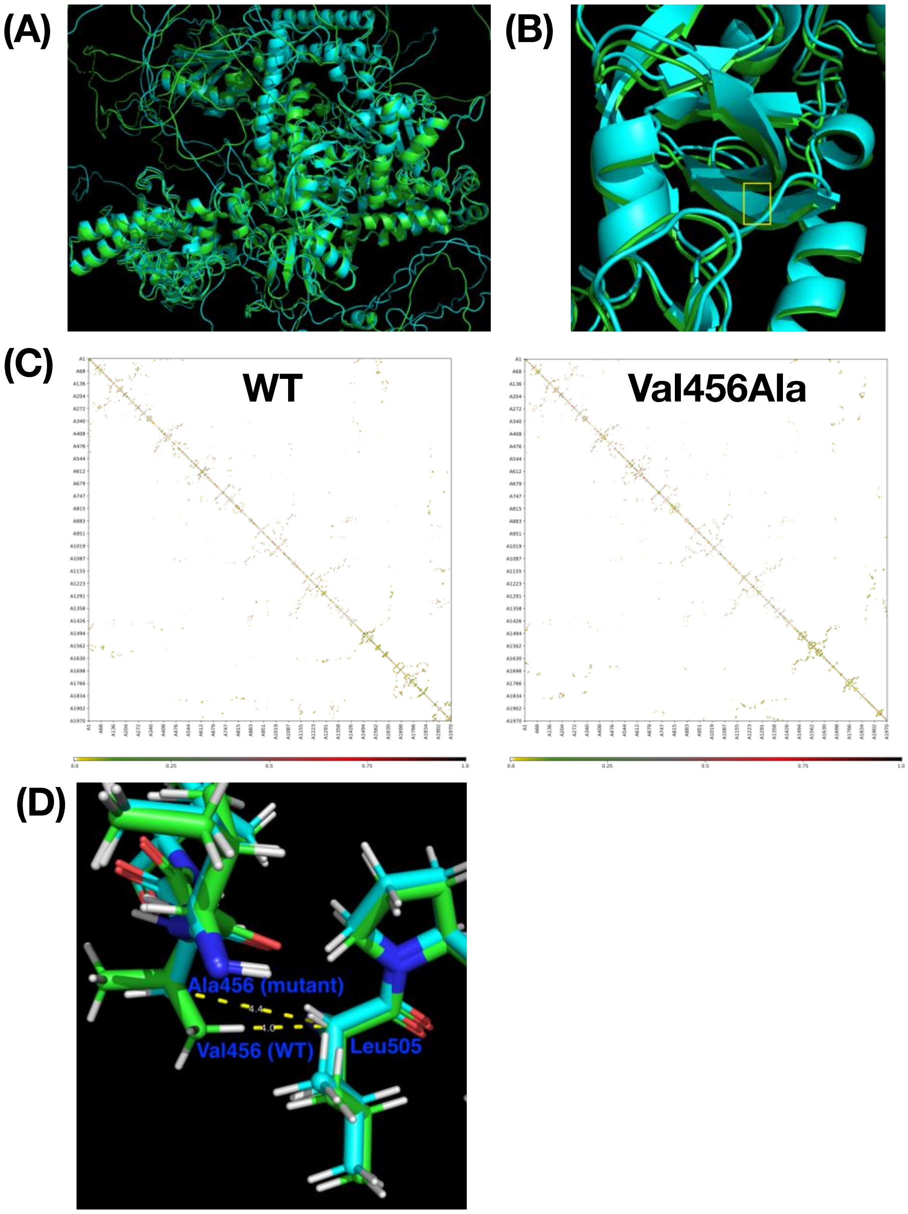

Figure S1. Dynamic molecular simulations observe no gross structural changes predicted between wild type and p.V456A mutant. (A) Cartoon representation of the predicted three-dimensional structures of the wild-type (green) and mutant (blue) proteins. (B) Cartoon representation focusing on the area surrounding the mutated residue (yellow box). (C) Amino acid contact maps for the wild-type protein and the p.Val456Ala mutant as predicted by the CABS-flex 2.0 server [28]. (D) Stick representation showing a close-up view of residue 456 and a nearby hydrophobic residue, Leu505. The yellow dotted lines represent the distance in angstroms between Leu505 and the two versions of residue 456.

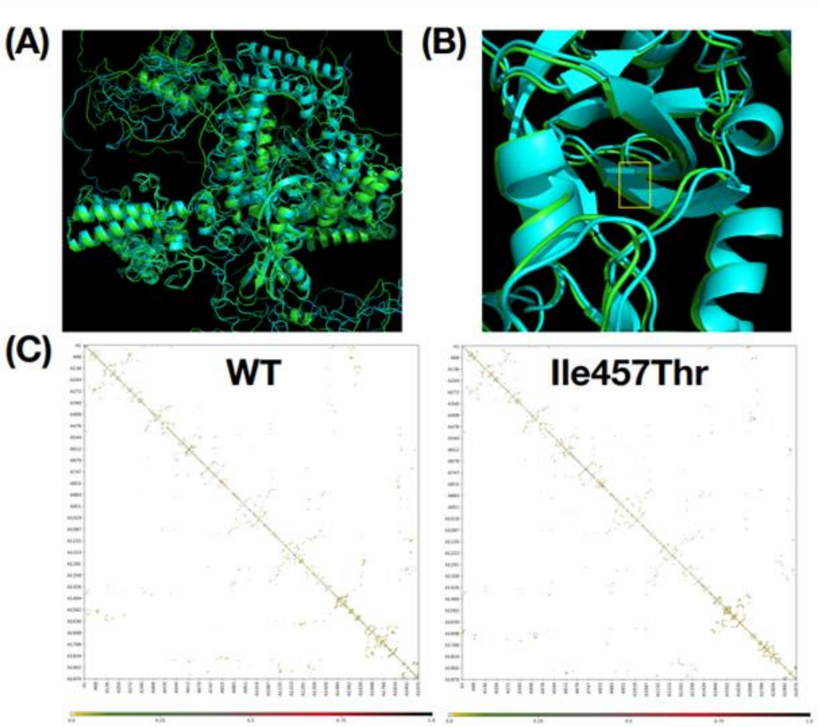

Figure S2. Dynamic molecular simulations observe no gross structural changes predicted between wild type and p.I457T mutant. (A) Cartoon representation of the predicted three-dimensional structures of the wild-type (green) and mutant (blue) proteins. (B) Cartoon representation focusing on the area surrounding the mutated residue (yellow box). (C) Amino acid contact maps for the wild-type protein and the p. Ile467Thr mutant as predicted by the CABS-flex 2.0 server [28].

Table S1. Variants detected in our patient reported as strong candidate ASD genes according to SFARI ASD genes.

Gene	Disorder in OMIM	Inheritance	Variant	Exon	Effect	Others
RERE	Neurodevelopmental disorder with or without anomalies of the brain, eye, or heart,616975,AD	mat	NM_001042681.2:c.4519A>G;NP_001036146.1:p.Ile1507Val; 1:8355567	22	Missense	SFARI score 1S. CADD=23; 3/5 predicted damaging; gnomAD allele frequency: 0.0001057
ZMYND11	Mental retardation, 30,616083,AD	mat	NM_006624.5:c.824A>T;NP_006615.2:p.Tyr275Phe; 10:240963	9	Missense	SFARI score 2. CADD=23; 1/5 predicted damaging; gnomAD allele frequency: 0.00005913
HERC2	Mental retardation, 38,615516,AR	Pat	NM_004667.5:c.6385C>T;NP_004658.3:p.Arg2129Cys;15:28214246	41	Missense	SFARI score 2. CADD=25.5; 3/5 predicted damaging; gnomAD allele frequency: 0.0006241

Legend: AD (autosomal dominant), AR (autosomal recessive), mat (maternal), pat (paternal). There were no *de novo* variants in the SFARI genes. There were 3 variants in genes with high scores according to SFARI (i.e. RERE, ZMYND11 and HERC2). Each of these are paternally inherited, while both parents in our study family were neurotypical, thus paternal transmission was not supported by pedigree structure. Furthermore, *HERC2* is associated with autosomal recessive transmission and thus unlikely to be causative, while *RERE* and *ZMYND11* are OMIM genes with autosomal dominant pattern. These variants are reported at low frequency but identified in numerous cases among the healthy gnomad dataset. For example, 16 counts as allele frequency 0.0001057 for the variant in *RERE*; 9 counts as the allele frequency 0.00005913 for the variant in *ZMYND11*; and 95 counts as frequency 0.0006241 for the variant in *HERC2*. The SFARI database can be accessed at https://gene.sfari.org/database/human-gene/.
